# AL Amyloidosis Patients Continue to Benefit from HDCT/ASCT Consolidation in the Daratumumab Era

**DOI:** 10.3390/jcm15041564

**Published:** 2026-02-16

**Authors:** Julia Bee, Inna Shaforostova, Michèle Hoffmann, Martina Bertschinger, Katja Seipel, Ulrike Bacher, Thomas Pabst

**Affiliations:** 1 Department of Medical Oncology, Bern University Hospital, University of Bern, 3010 Bern, Switzerland; julia.bee@students.unibe.ch (J.B.); innaivanovna.shaforostova@insel.ch (I.S.); michele.hoffmann@insel.ch (M.H.); martina.bertschinger@insel.ch (M.B.);; 2 Department of Clinical Chemistry, Inselspital, Bern University Hospital, University of Bern, 3010 Bern, Switzerland; 3 Department of Hematology, Bern University Hospital, University of Bern, 3010 Bern, Switzerland; veraulrike.bacher@insel.ch; 4 Center for Hemato-Oncology, University Cancer Center, 3010 Bern, Switzerland

**Keywords:** high-dose chemotherapy (HDCT), autologous stem cell transplantation (ASCT), daratumumab, AL amyloidosis, prognosis

## Abstract

**Background/Objectives**: Previous studies suggested superior outcomes for AL amyloidosis patients eligible for consolidation with high-dose chemotherapy (HDCT) and autologous stem cell transplantation (ASCT) compared to patients who did not receive these therapies. However, data are limited due to disease rarity, differing patient selection, and evolving treatment algorithms. Following the introduction of daratumumab, which improved outcomes in AL amyloidosis patients, it remains unclear whether HDCT/ASCT still confers additional benefit. **Methods**: Our retrospective, single-center study aimed to compare patients diagnosed between January 2003–December 2024 and consolidated in first remission with HDCT/ASCT vs. without HDCT/ASCT, both before and within the era of CD38-targeting daratumumab. **Results**: In our cohort of 106 AL systemic amyloidosis patients, 57 patients underwent HDCT/ASCT after induction therapy, while 49 had chemoimmunotherapy regimens alone. The two groups differed at initial diagnosis by age (*p* = 0.0028), renal function (eGFR, *p* = 0.0054), Troponin T levels (*p* < 0.0001) and NT-proBNP (*p* = 0.038). Patients treated with HDCT/ASCT had considerably better outcomes than patients without HDCT/ASCT. The median overall survival (OS) was 157 vs. 36 months (*p* < 0.0001), and median progression-free survival (PFS) was 81 vs. 24 months (*p* < 0.0001). Daratumumab was given to 45 patients (41.7%) during first line treatment, and patients were divided into additional subgroups: HDCT/ASCT ± daratumumab and chemotherapy ± daratumumab. OS and PFS were longer in patients treated with HDCT/ASCT, regardless of whether daratumumab was added to induction. The 5-year OS was 88%/86% in patients treated with HDCT with/without daratumumab and 37%/47% in the chemoimmunotherapy group with/without daratumumab (n.s.). The 5-year PFS in patients receiving HDCT was 63%/78% and in the group without HDCT/ASCT 38%/22% each with/without daratumumab. **Conclusions**: Thus, regardless of daratumumab use during induction treatment, our results strongly suggest that patients with AL amyloidosis undergoing HDCT/ASCT consolidation achieve superior PFS and OS compared with those treated with chemoimmunotherapy alone.

## 1. Introduction

Immunoglobulin light chain (AL) amyloidosis is a rare clonal plasma cell disorder characterized by the extracellular deposition of misfolded, light chain-derived amyloid fibrils in various organs. AL amyloidosis is one of the most common forms of systemic amyloidosis, besides transthyretin (ATTR) amyloidosis, accounting for approximately 60–85% of all cases [1]. The estimated incidence is around 10 patients per million individuals per year, making it nearly five times less common than multiple myeloma. Cardiac and renal involvement are most frequently seen (heart: 60–75% of patients; kidneys: 70% of patients), often resulting in significant organ dysfunction. Less commonly, the nervous system, liver, gastrointestinal tract, spleen, skin, tongue, and joints are involved. The pathogenic light chains are produced by clonal CD38-positive plasma cells, similar to those seen in multiple myeloma. The deposited light chains are predominantly of the lambda isotype, with a lambda-to-kappa ratio of approximately 3:1 [2,3,4,5,6,7,8,9].

The causes of protein misfolding in amyloid light-chain amyloidosis include patient-specific somatic mutations in the immunoglobulin light chain variable domain, particularly within the complementarity-determining regions, which destabilize the native fold and increase protein dynamics [10].

Light-chain (AL) and transthyretin (ATTR) amyloidosis differ substantially in their clinical presentation, organ involvement, and prognosis. AL amyloidosis typically affects younger patients, progresses rapidly, and is frequently associated with multiorgan involvement, including the kidneys, liver, and nervous system, in addition to the heart. Cardiac involvement in AL amyloidosis is characterized by severe diastolic dysfunction, elevated cardiac biomarkers, and direct myocardial toxicity from circulating light chains, resulting in high early mortality. In contrast, ATTR amyloidosis predominantly affects older individuals, often presents as isolated cardiac disease, and follows a more indolent course with slowly progressive heart failure. Non-cardiac manifestations such as carpal tunnel syndrome and spinal stenosis are more commonly observed in ATTR amyloidosis and may precede cardiac symptoms [11].

Within AL amyloidosis a distinction should be made between primary AL amyloidosis and AL amyloidosis associated with multiple myeloma. This classification is based primarily on the plasma cell burden in the bone marrow (>10% vs. ≤10%) and the presence or absence of SLiM-CRAB criteria (hypercalcemia, renal dysfunction, anemia, and bone lesions). Patients with AL amyloidosis and more than 10% bone marrow plasma cells, even in the absence of SLiM-CRAB features, are often referred to as having AL amyloidosis with plasma cell multiple myeloma (AL-PCMM). These patients generally have a clinical course and prognosis comparable to those with overt myeloma and concurrent amyloidosis [12].

The outcome of AL amyloidosis patients largely depends on the organ involvement, particularly cardiac involvement, as well as the timing of the initial diagnosis and cytogenetic risk group. The revised Mayo risk prediction model, which incorporates NT-proBNP and troponin T levels, is used to estimate prognosis based on the extent of cardiac involvement—one of the most critical determinants of outcome in AL amyloidosis. Without treatment, patients with AL amyloidosis have an average survival of around 13 months after initial diagnosis. If heart failure is already present, overall survival is reduced to around four months [2,3,4,5,13,14,15,16,17,18]. A recent study from Khwaja et al. reported a new staging model where they combined the Mayo risk prediction model with the longitudinal strain (LS) by echocardiography. LS is a sensitive marker for left ventricular dysfunction and seems to be an independent predictive marker for poor outcome in patients with AL amyloidosis and is used to better define the high-risk groups IIIb and IIIc [19]. Besides cardiac dysfunction, renal involvement contributes substantially to morbidity and may limit therapeutic options. Renal staging is based on estimated glomerular filtration rate (eGFR) and the degree of proteinuria, with three stages defined by specific thresholds [17,20].

Until recently, the standard treatment for AL amyloidosis consisted of bortezomib-based chemotherapy-regimens, most commonly a combination of cyclophosphamide, bortezomib, and dexamethasone (CyBorD). However, the ANDROMEDA trial demonstrated that the addition of daratumumab—a monoclonal antibody targeting CD38—to the CyBorD backbone significantly improved complete response rates, organ response rates, and progression-free survival (PFS) compared to CyBorD alone. Consequently, daratumumab is now combined with CyBorD (Dara-CyBorD) as first-line therapy [2,6,18].

High-dose chemotherapy (HDCT) with melphalan followed by autologous hematopoietic stem cell transplantation (ASCT) has been used since the 1990s as a consolidation treatment in selected patients with AL amyloidosis who have responded favorably to initial therapy. In multiple myeloma, this is the standard therapy for fit patients since the late 1990’s [21]. Several studies have shown that patients with AL amyloidosis have an improved outcome with HDCT/ASCT compared to chemotherapy alone [18,22,23,24,25,26,27]. Approximately 70% of patients receiving HDCT/ASCT achieved very good partial remission (VGPR) or complete remission (CR) with median survival reaching up to 15 years in those attaining CR. However, only about 6.2% of patients with AL amyloidosis are considered fit enough to tolerate HDCT/ASCT representing the first major limitation of this intensive treatment strategy [2,28,29]. At the same time, application or, rather, tolerance of HDCT/ASCT treatment itself is a favorable prognostic factor.

The second major limitation is the high treatment-related mortality associated with HDCT/ASCT, which has been reported to reach more than 40%, in some studies, particularly among patients with high-risk factors [30,31,32]. This highlights the clinical importance of careful patient selection based on performance status, age, cardiac and renal function, and extent of organ involvement [2,3,4,5,22,28,32].

Most studies evaluating the efficacy of HDCT/ASCT in AL amyloidosis were conducted during the era when proteasome inhibitor-based regimens were the primary treatment option. There is limited data on whether HDCT/ASCT continues to improve patient outcomes in the current era, characterized by the availability of more effective CD38-targeted therapies. Therefore, we conducted a retrospective analysis to assess the long-term outcomes and role of HDCT/ASCT in a cohort of patients with AL amyloidosis and to evaluate the impact of HDCT/ASCT in the context of modern treatment regimens including daratumumab.

## 2. Methods

### 2.1. Patients

We performed a single-center retrospective study of a cohort of consecutive patients with AL amyloidosis who were diagnosed and treated at the University Hospital in Bern, Switzerland, between 2003 and 2024. The diagnosis was confirmed by biopsy with Congo red staining and double refraction revealing amyloid composed of monoclonal immunoglobulin light chains. Patients with AL amyloidosis were included, also those with secondary amyloidosis due to multiple myeloma or those with only localized disease (amyloid tumor). However, only patients with systemic primary or secondary AL amyloidosis were included in the final analysis. Organ involvement was assessed using the revised Mayo risk prediction model with Mayo Stage I–IV for cardiac involvement and Stage I–III for renal involvement [17]. The deadline for recruitment of follow-up data was 31 May 2025.

Patients were divided into two groups: one group received HDCT/ASCT with melphalan conditioning as described before, while the second received conventional treatment without HDCT/ASCT. Only patients with ECOG ≤ 2, absence of advanced organ dysfunction, and limited multiorgan involvement were considered eligible for HDCT/ASCT. The melphalan dose was generally 200 mg/m^2^. In a few patients with significantly reduced eGFR, the dose was reduced to 100–170 mg/m^2^. In a second step, these two groups were separated into four subgroups: HDCT/ASCT with or without daratumumab, and chemoimmunotherapy (cyclophosphamide, bortezomib, dexamethasone) with or without daratumumab. Daratumumab was first administered at our center in March 2018.

For further analysis, we divided the patients into a low-risk and a high-risk group based on cytogenetical abnormalities in fluorescence in situ hybridization (FISH) analysis. We defined “high-risk” as the presence of two or more of the following abnormalities: t(4;14), t(14;16), t(14;20), del17p, hyperdiploidy, and gain of 1q [33]. All other cytogenetic subgroups by FISH were classified as low-risk.

### 2.2. Statistical Analysis

This observational study aims to identify any significant differences in OS and PFS between patients who received HDCT/ASCT and those who received conventional chemoimmunotherapy. The two treatment groups were analyzed in terms of the primary outcome of OS and the secondary outcomes of PFS, remission status, and the relationship between the outcomes and FISH risk groups. OS was defined as the time between initial diagnosis and the latest follow-up or death. PFS was defined as the time between initial diagnosis and first progression or relapse, or death or last follow-up if patients remained in remission. The classification of remission status was based on the response criteria of the International Society of Amyloidosis [34]. Microsoft Excel was primarily used for data evaluation. Baseline characteristics were compared using a t-test or chi-squared test. Kaplan–Meier curves were generated with GraphPad Prism 10.4.1. The *p*-values of the survival curves were assessed using the log-rank test.

## 3. Results

### 3.1. Baseline Characteristics of the Patients

We identified 108 patients with AL amyloidosis in our center within the study period, including 2 patients with localized amyloidosis and 106 with systemic primary or secondary AL amyloidosis. Among these, 54 patients had MM-associated AL amyloidosis (AL/MM), and 52 had primary systemic AL amyloidosis. Overall, 106 patients were included in the final analysis. A total of 57 patients received HDCT/ASCT, while the remaining 49 were treated with conventional chemoimmunotherapy. Several baseline characteristics differed between the two treatment groups. Patients in the HDCT/ASCT group were slightly younger at diagnosis (median 62 vs. 64 years; ***p* = 0.0032**). They also exhibited lower disease severity, reflected by less adverse cardiac biomarker levels—troponin T (29 µg/L vs. 43 µg/L; ***p* < 0.0001**) and NT-proBNP (1034 ng/L vs. 3049.5 ng/L; ***p* = 0.038**)—corresponding to lower revised Mayo stage. In addition, renal function was better in the HDCT/ASCT group, as indicated by higher eGFR values (78 mL/min vs. 60 mL/min; ***p* = 0.0054**). The other baseline characteristics were comparable between the two groups (Table 1). At diagnosis, 35 patients (61.4%) in the HDCT/ASCT group and 29 (56.9%) in the group without HDCT/ASCT showed cardiac involvement (*p* = 0.6316). The extent of renal dysfunction was lower in the HDCT/ASCT patients than in those without HDCT/ASCT (19 (33%) vs. 25 (49%) patients, *p*-value 0.09). Other organ involvements are listed in Table 1.

#### 3.1.1. Outcome in the Whole Study Population

**Figure 1:**
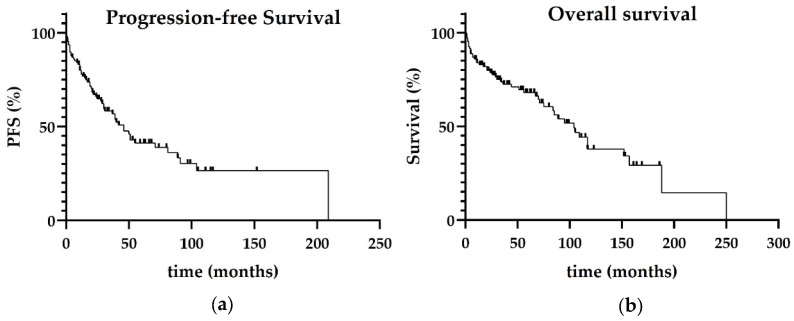
(**a**) Progression-free survival and (**b**) overall survival of the entire cohort.

The median follow-up period for the study cohort was 41 months (range 1–250). The median OS of this cohort was 104 months (range 1–250) and the median PFS was 46 months (range 0.1–209, see Figure 1).

During the follow-up period, 14 patients (14.6%) in the HDCT/ASCT group and 34 patients (66.7%, ***p* < 0.0001**) in the group (without HDCT/ASCT died (see Table 2)).

**Figure 2:**
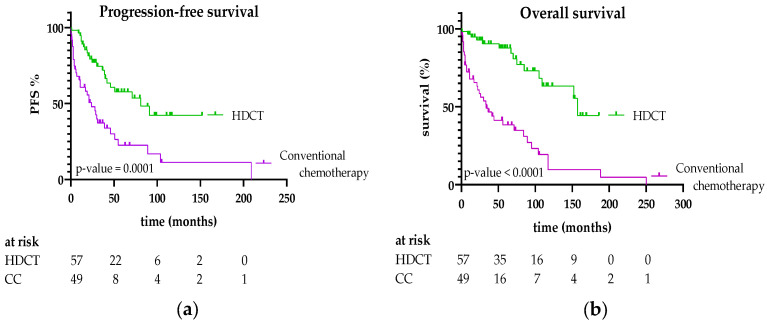
(**a**) PFS and (**b**) OS of patients treated with HDCT/ASCT or conventional chemoimmunotherapy.

Patients receiving HDCT/ASCT had significantly longer OS and PFS compared with those without HDCT/ASCT. Median OS was 157 vs. 36 months, with 5-year OS rates of 87% vs. 41% (***p* < 0.0001**), and median PFS was 81 vs. 24 months, with 5-year PFS rates of 57% vs. 22% (***p* < 0.001**). Survival curves are shown in Figure 2.

The treatment related mortality (defined as death in the first 100 days after ASCT) in patients treated with HDCT/ASCT was 6.1% in the group without daratumumab and 0% in the group with additional daratumumab.

In multivariate analysis, HDCT/ASCT remained independently associated with improved PFS and OS (Table 3).

#### 3.1.2. Outcomes in Comparison to Daratumumab Use

**Figure 3:**
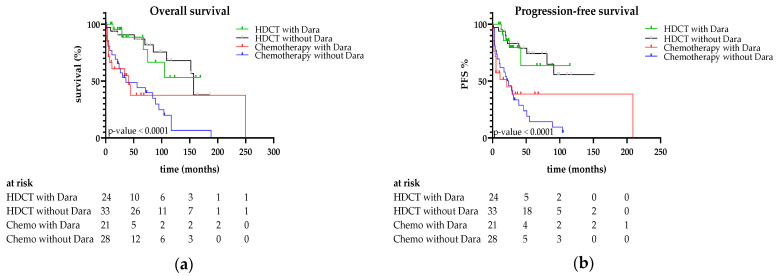
(**a**) PFS and (**b**) OS of patients treated with HDCT/ASCT ± daratumumab versus chemoimmunotherapy ± daratumumab.

In an additional analysis, the patients were divided into four groups: HDCT/ASCT ± daratumumab and without HDCT/ASCT ± daratumumab. Figure 3 shows the survival curves of these four groups. Median overall survival has not yet been reached in patients treated with HDCT/ASCT, with or without daratumumab. Five-year OS was 88% with HDCT/ASCT plus daratumumab and 86% without, compared with 37% and 47% in patients without HDCT/ASCT (***p* < 0.0001**). PFS was significantly longer in the HDCT/ASCT groups regardless of daratumumab, with median PFS not yet reached. Five-year PFS rates were 63% and 78% with HDCT/ASCT with and without daratumumab, versus 38% and 22% without HDCT/ASCT (***p* < 0.0001**).

#### 3.1.3. Outcomes by Cytogenetic Risk Group

**Figure 4:**
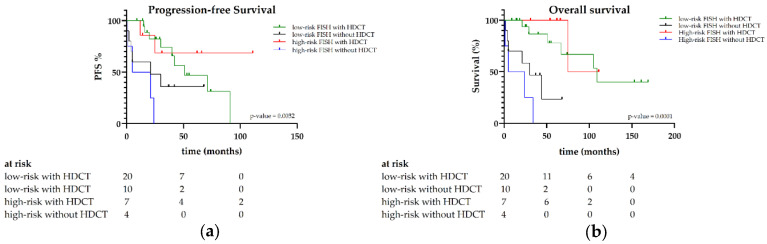
(**a**) PFS and (**b**) OS according to cytogenetic risk groups by FISH in the HDCT/ASCT patients versus patients without HDCT/ASCT.

Of the 41 patients who received FISH testing, 19 (46.3%) demonstrated the t(11;14) translocation, the most common cytogenetic abnormality in AL amyloidosis. Therapy in patients receiving HDCT/ASCT appears to be superior for achieving CR, with 77.2% versus 25.5% in patients without HDCT/ASCT (***p* < 0.0001**, Table 2). The analysis of OS by FISH risk groups showed superior survival with HDCT/ASCT, regardless of whether patients had high- or low-risk FISH markers (***p* < 0.001**). Five-year OS was 77% and 100% with HDCT/ASCT versus 23% and 0% in patients without HDCT/ASCT, for low- and high-risk cytogenetic groups by FISH, respectively (***p* < 0.001**). PFS also favored HDCT/ASCT, with five-year PFS of 46% and 68% versus 36% and 0% for low- and high-risk groups in the chemoimmunotherapy (without HDCT/ASCT) group (***p* = 0.0032**, Figure 4).

## 4. Discussion

This retrospective single-center study demonstrates a clear advantage in both OS and PFS for patients with AL amyloidosis treated with HDCT/ASCT compared with those without HDCT/ASCT. These results are consistent with previous reports, including an earlier study from our institution, confirming the superior prognosis of AL amyloidosis patients treated with HDCT/ASCT [3,16,18,19]. In real-world practice, only a minority of AL amyloidosis patients qualify for HDCT/ASCT. In contrast, more than half of the patients in our cohort underwent HDCT/ASCT, representing a higher-than-typical proportion. This likely reflects both selection and referral biases, as patients are frequently transferred from local hospitals only if deemed fit for HDCT/ASCT. Eligibility for HDCT/ASCT itself is a favorable prognostic factor and may partially explain the more favorable outcomes observed in the HDCT/ASCT group [1,20]. Nevertheless, our data clearly demonstrate that eligible patients derive substantial benefit from HDCT/ASCT.

Cardiac involvement based on NT-proBNP levels, one of the strongest predictors of early mortality, was significantly more common in the group without HDCT/ASCT. Despite this, transplanted patients achieved superior OS and PFS compared with those receiving conventional therapy, highlighting the efficacy of high-dose therapy even in patients with significant organ involvement. The survival benefit of HDCT/ASCT persisted after the introduction of daratumumab-containing induction therapy. Importantly, our data show that HDCT/ASCT continues to outperform chemoimmunotherapy alone even in the era of daratumumab, indicating that monoclonal antibody-based induction does not replace the added value of HDCT/ASCT for fit patients. Unlike the ANDROMEDA trial, our study did not directly compare chemoimmunotherapy with versus without daratumumab. Therefore, no definitive conclusion can be drawn regarding the additional benefit of daratumumab in this setting, likely due to the small sample size, retrospective design, and limited follow-up [5]. Given that cardiac and renal involvement are the principal drivers of mortality in AL amyloidosis, early and effective plasma cell–directed therapy is critical. Regimens such as Dara-CyBorD induce rapid hematologic responses by eliminating amyloidogenic plasma cell clones, leading to improvements in organ function and survival, while HDCT/ASCT provides deeper and more durable clonal eradication through sustained suppression of pathogenic light-chain production. Cytogenetic analysis revealed that HDCT/ASCT patients had better outcomes regardless of high- or low-risk abnormalities. The frequency of t(11;14) in our cohort (46.3%) was consistent with previous reports [17,24].

Finally, our findings are consistent with the previous study by Raschle et al., confirming the role of HDCT/ASCT in eligible patients with AL amyloidosis and strengthening the evidence through a larger cohort (63 vs. 108 patients) and longer follow-up (31 vs. 57 months) [3].

Several limitations of our study must be acknowledged. The retrospective design introduces inherent biases, sample size remains limited for subgroup comparisons, follow-up for patients receiving daratumumab-containing regimens is still immature, and the monocentric nature of this tertiary referral center study may limit generalizability due to referral- and selection-related biases. In particular, patients who received HDCT/ASCT had more favorable baseline characteristics regarding age, renal function, and cardiac biomarker levels, which may have contributed to the improved PFS and OS observed in this group. Patients in the non-HDCT/ASCT group had generally less favorable characteristics including older age and poorer organ function. Although we performed a multivariate analysis to adjust these differences, the residual confounding remains. In addition, the revised Mayo staging could only be determined in a subset of patients with cardiac and renal involvement, which limits the interpretability of comparisons between the treatment groups. Finally, patients with AL/MM have distinct biological characteristics and may exhibit different treatment responses, representing a potential confounding factor in our analysis.

In conclusion, our data suggest that HDCT/ASCT provides substantial and durable survival benefits, remaining the most effective consolidation approach for patients with AL amyloidosis, even in the era of daratumumab-containing induction therapy. While emerging therapies that are adapted from multiple myeloma are likely to reshape future treatment strategies, HDCT/ASCT currently retains its role as the standard of care for eligible individuals with AL amyloidosis. Future studies with larger cohorts and longer follow-up are needed to refine which patients may achieve sufficient disease control with daratumumab-based induction alone and which high-risk subgroups, particularly those with adverse cytogenetic features, derive the greatest benefit from HDCT/ASCT.

## Figures and Tables

**Table 1:** Baseline characteristics of the study population.

Parameter	HDCT/ASCT in Total	Without HDCT/ASCT in Total	*p*-Value
Number of patients	57	49	
Age at initial diagnosis, years, median (range)	62 (31–74)	64 (45–86)	**0.0032**
Sex, male/female	28/29	30/19	0.1805
Daratumumab-based induction, *n*	24 (42.11%)	21 (41.28%)	0.9221
Troponin T, ug/L, median (range)	29 (0.01–125)	44 (8–399)	**<0.0001**
NT-proBNP, ng/L, median (range)	1034 (49–13,230)	3100 (63–82,342)	**0.0382**
β2-microglobulin, mg/L, median (range)	2.6 (1.3–11.2)	3.8 (0.4–19.4)	0.0614
Albumin, g/L, median (range)	32 (4–42)	31 (11–43)	0.5031
Paraprotein value, g/L			
IgG, median (range)	6.6 (1.7–60.5)	9.8 (1.7–22.4)	0.4621
IgA, median (range)	0.73 (0.1–18)	1.25 (0.2–4.9)	0.0623
IgM, median (range)	0.4 (0.04–2.5)	0.58 (0.1–36.9)	0.1421
Proteinuria, g/L, median (range)	0.5 (0.04–27.4)	0.71 (0.04–14.9)	0.6091
Hemoglobin, g/L, median (range)	131 (88–159)	124 (80–173)	0.1550
Leucocytes, G/L, median (range)	6.8 (4.7–191)	7.8 (4.4–347)	0.6921
Platelets, G/L, median (range)	257 (5.6–587)	290 (6.9–834)	0.2965
Creatinine, umol/L, median (range)	84 (42–290)	86 (46–481)	0.0692
eGFR, mL/min, median (range)	78 (22–112)	60 (14–91)	**0.0054**
Plasma cells in bone marrow, %, median (range)	15 (4–90)	12 (5–90)	0.7561
Number of involved organs			
1	17	15	0.6543
2	30	10	**0.0004**
3	3	16	**0.0004**
4 or more	7	8	0.6094
Organ involvement			
cardiac	35 (61.4%)	29 (56.9%)	0.6316
renal	19 (33.33%)	25 (49.02%)	0.0977
gastrointestinal	16 (28.1%)	20 (39.22%)	0.2200
liver	1 (1.75%)	4 (7.84%)	0.1328
peripheral nervous system	0 (0%)	1 (1.96%)	-
Mayo stage			
cardiac: data in 35 of 38 patients available			
Mayo I	0	0	-
Mayo II	10	5	0.2433
Mayo III	15	13	0.8951
Mayo IV	0	3	-
renal: data in 16 of 22 patients available			
I	6	4	0.0627
II	4	12	0.188
III	2	5	0.6292
Cytogenetics, data in 41 of 108 patients available			
normal cytogenetics	3	2	1.000
t(11;14)	10	9	1.000
t(4;14)	7	3	0.3281
t(14;16)	6	3	0.4953
del17p	0	1	0.4722
gain1q	10	2	**0.0317**
loss1p	1	1	1.000
del13p	11	1	**0.0047**
Hyperdiploidy	1	3	0.3418
others	12	4	0.0624

Abbreviations: NT-proBNP = N-terminal prohormone of brain natriuretic peptide, IgG = Immunoglobulin G, IgA = Immunoglobulin A, IgM = Immunoglobulin M, eGFR = estimated glomerular filtration rate, Mayo stage = modified Mayo Classification 2013.

**Table 2:** Comparison of treatment groups regarding outcome.

Parameter	HDCT/ASCT in Total	Without HDCT/ASCT in Total	*p*-Value	HDCT/ASCT with Daratumumab	Without HDCT/ASCT with Daratumumab	*p*-Value
Follow-up, median, months	59	26	**0.0076**	30	16	**0.0416**
Best response to therapy						
Complete response	44 (77.2%)	13 (25.5%)	**<0.0001**	20	7	**0.0034**
Very good partial response	7 (12.3%)	7 (13.7%)	0.4750	1	6	**0.0313**
Others *	0 (0%)	16 (31.4%)	-	0	6	-
Progression or relapse	19 (33.3%)	20 (39.2%)	0.5250	7 (29.17%)	6 (28.57%)	0.96
Death	14 (24.6%)	34 (66.7%)	**<0.0001**	5 (20.83%)	12 (57.14%)	**0.015**

* Partial response; stable disease.

**Table 3:** Multivariate analysis of factors associated with PFS and OS in amyloidosis.

	PFS			OS		
Predictors	HR	95% CI	*p*-Value	HR	95% CI	*p*-Value
Age, years	0.97	0.93–1.03	0.31	0.98	0.92–1.04	0.56
NT-proBNP, ng/L	1.00	1.00	0.10	1.00	1	**0.02**
eGFR, mL/min	0.99	0.98–1.01	0.56	0.99	0.97–1.01	0.47
HDCT/ASCT vs. without HDCT/ASCT	0.21	0.09–0.50	**0.0004**	0.17	0.06–0.48	**0.0008**

Abbreviations: NT-proBNP: N-terminal pro-B-type natriuretic peptide; eGFR: estimated Glomerular Filtration Rate; HDCT/ASCT: High dose chemotherapy/autologous stem cell transplant; PFS: progression-free survival; OS: Overall survival.

## Data Availability

The data presented in this study are available upon request from the corresponding author.
